# Plan quality and treatment efficiency assurance of two VMAT optimization for cervical cancer radiotherapy

**DOI:** 10.1002/acm2.14050

**Published:** 2023-05-29

**Authors:** Sijuan Huang, Xiuying Mai, Hongdong Liu, Wenzhao Sun, Jinhan Zhu, Jinlong Du, Xi Lin, Yujie Du, Kang Zhang, Xin Yang, Xiaoyan Huang

**Affiliations:** ^1^ Department of Radiation Oncology, Sun Yat‐sen University Cancer Center State Key Laboratory of Oncology in South China, Collaborative Innovation Center for Cancer Medicine Guangdong Key Laboratory of Nasopharyngeal Carcinoma Diagnosis and Therapy Guangzhou Guangdong China; ^2^ School of Biomedical Engineering Guangzhou Xinhua College Guangzhou Guangdong China; ^3^ United Imaging Healthcare Shanghai China

**Keywords:** cervical cancer, delivery time, fluence map optimization (FMO), gamma pass rate (GPR), modulation complexity score (MCS), plan difference, stochastic platform optimization (SPO)

## Abstract

To investigate the difference of the fluence map optimization (FMO) and Stochastic platform optimization (SPO) algorithm in a newly‐introduced treatment planning system (TPS). Methods: 34 cervical cancer patients with definitive radiation were retrospectively analyzed. Each patient has four plans: FMO with fixed jaw plans (FMO‐FJ) and no fixed jaw plans (FMO‐NFJ); SPO with fixed jaw plans (SPO‐FJ) and no fixed jaw plans (SPO‐NFJ). Dosimetric parameters, Modulation Complexity Score (MCS), Gamma Pass Rate (GPR) and delivery time were analyzed among the four plans. Results: For target coverage, SPO‐FJ plans are the best ones (*P* ≤ 0.00). FMO plans are better than SPO‐NFJ plans (*P* ≤ 0.00). For OARs sparing, SPO‐FJ plans are better than FMO plans for mostly OARs (*P* ≤ 0.04). Additionally, SPO‐FJ plans are better than SPO‐NFJ plans (*P* ≤ 0.02), except for rectum V45Gy. Compared to SPO‐NFJ plans, the FMO plans delivered less dose to bladder, rectum, colon V40Gy and pelvic bone V40Gy (*P* ≤ 0.04). Meanwhile, the SPO‐NFJ plans showed superiority in MU, delivery time, MCS and GPR in all plans. In terms of delivery time and MCS, the SPO‐FJ plans are better than FMO plans. FMO‐FJ plans are better than FMO‐NFJ plans in delivery efficiency. MCSs are strongly correlated with PCTV length, which are negatively with PCTV length (*P* ≤ 0.03). The delivery time and MUs of the four plans are strongly correlated (*P* ≤ 0.02). Comparing plans with fixed or no fixed jaw in two algorithms, no difference was found in FMO plans in target coverage and minor difference in Kidney_L Dmean, Mu and delivery time between PCTV width≤15.5 cm group and >15.5 cm group. For SPO plans, SPO‐FJ plans showed more superiority in target coverage and OARs sparing than the SPO‐NFJ plans in the two groups. Conclusions: SPO‐FJ plans showed superiority in target coverage and OARs sparing, as well as higher delivery efficiency in the four plans.

## 1 INTRODUCTION

Volumetric modulated arc therapy (VMAT) is a widely adopted delivery radiotherapy techniques in clinics. It simultaneously integrates multi‐leaf collimator (MLC) field shape modulation with gantry speed and dose rate variations among adjacent fields or gantry angles. With more degrees of freedom during treatment, VMAT plans provide better dose distribution to targets and little dose to organs at risks (OARs), and deliver plans more efficiently than static beam intensity modulated radiotherapy (IMRT).^1^, ^2^ The VMAT technique has become clinically and commercially available in all kinds of treatment planning system (TPS) and linear accelerators. However, there are many differences in machines^3^ and optimization algorithms,^4^ for example, the method of dose rate control,^5^ and jaw tracking technique,^6^ etc.

In 2021, a newly designed CT‐integrated linac named uRT‐linac 506C (United Imaging Healthcare [UIH] Co., Shanghai, China) was installed and put in clinical operation in our center. The linac has 120 Multi Leaf Collimator (MLC). The central 20 cm is composed of 40 pairs MLC with a width of 0.5 cm, and the two ends 10 cm are respectively composed of 10 pairs MLCs with a width of 1 cm. The maximum travelling speed of the MLCs is 55 mm/s. UIH linac and treatment planning system offered volumetric modulated techniques named uARC. Two algorithms are provided for uARC optimization in the TPS: Fluence Map Optimization (FMO) and Stochastic Platform Optimization (SPO). The goal of both FMO and SPO algorithms is to provide initial apertures and monitor units (MUs) to the Control Point Optimization (CPO) algorithm, which optimizes MLC positions and MU for each control point to further improve the plan quality based on the objective functions. The FMO algorithm is a two‐step approach: Firstly, segments are separated for the initial beams with two intervals. 2D fluence map with specific resolution is created for each segment and the optimization engine is called to optimize the intensities of each grid of fluence maps according to the optimization objectives. It should be pointed out that the range of fluence maps for different arcs will be adaptively adjusted according to the target size in order to limit the irradiation range of different arcs so as to control the MLC leaves’ moving range in a single segment. Next, fluence maps of segments in a single beam are merged and combined to a whole fluence map in order to perform a leaf sequencing program to generate MLC positions and MU for each segment considering the MLC moving constraints and gantry speed. Default MLC moving constraints in a segment is determined by the default plan delivery time which was set to 90 seconds for a full 360°arc beam, that is, 4° per second. Further correction of MLC positions is performed to let the plan meet the machine executable constraint so that the generated plan can be used for the CPO stage.

For the SPO algorithm, segments are also separated with two intervals for the initial beams. In the initialization process, several adjacent segments are combined to one platform so that the whole arc is divided to several platforms. Different from the FMO algorithm that optimizes fluence map on each segment, the fluence maps are created and optimized on platforms in SPO algorithm, and a Simulated Annealing (SA) algorithm based leaf sequencing program is performed to generate MLC positions and MUs in platforms according to the optimized fluence map. The SA algorithm is an optimization algorithm that minimizes the discrepancies between the optimized fluence map and the sequenced fluence map. Machine delivery constraints are considered in and between platforms in the SA algorithm. This process guarantees that the sequencer starts with a series of deliverable arcs that do not violate any delivery constraints, so that the initial plan generated by SPO algorithm can be directly used for CPO stage, which is different to FMO method. The MLC leaves’ moving constraints between two adjacent control points is calculated and limited base on the gantry maximum rotation speed, that is, 6° per second, which means a higher gantry speed and a shorter leaf moving distance in segment than FMO method. The SPO algorithm keeps generating plans based on the maximum rotation speed of gantry, so it has a higher execution speed. In the contrast, due to the requirement of multiple iterations, the process of SPO algorithm is more complex and less efficient than that of the FMO algorithm. For target with large size, due to the limitation of strict leaf constraints of the SPO algorithm, it is necessary to narrow the exposure range by locking jaws, whereas the FMO algorithm can adjusted the exposure range adaptively so that it's not necessary to limit jaw position for FMO optimization.

**FIGURE 1:**
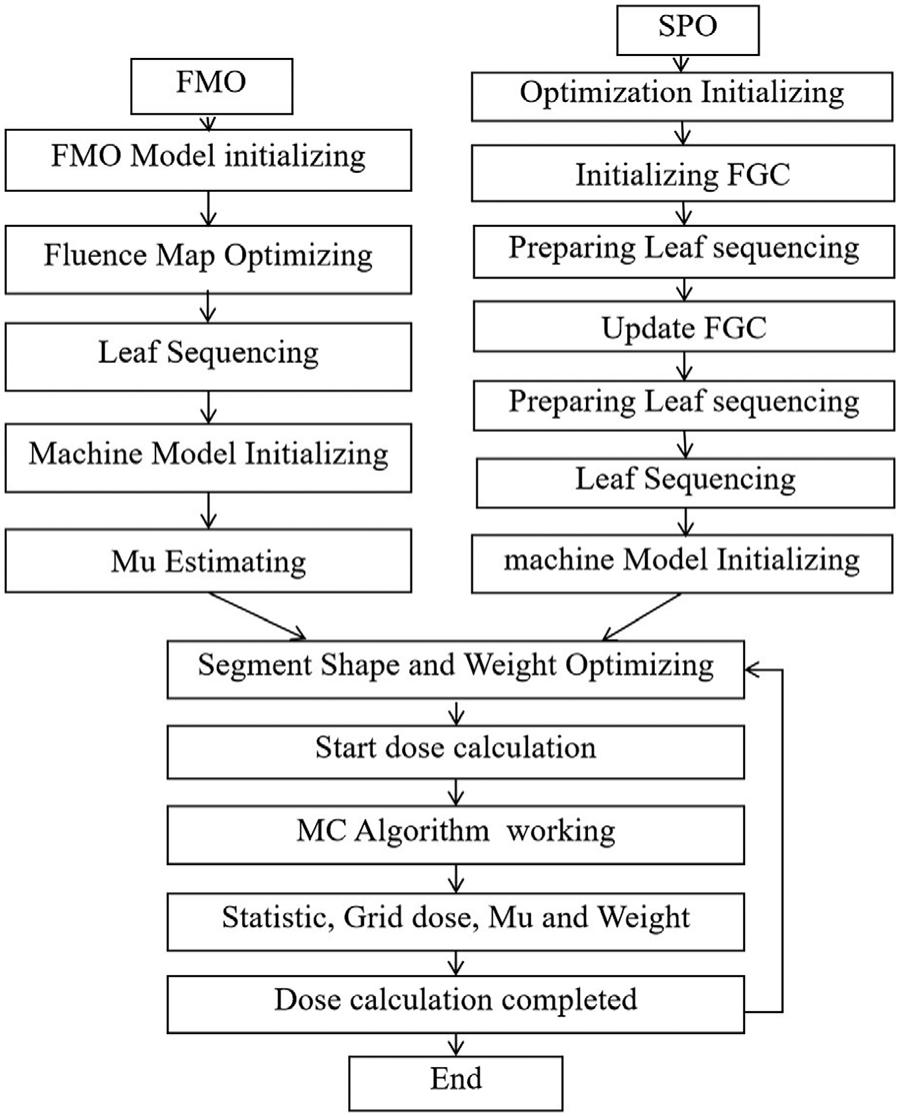
Comparison of FMO and SPO optimization algorithm (data from tps optimization console).

The stages of FMO and SPO process which were displayed on TPS interface when the optimization process was running is illustrated in Figure 1. As described above, the FMO algorithm starts from a FMO model initializing process and end with a MU estimating one, which determines an MU upper bound limitation in the next CPO stage. The SPO algorithm also starts with initialization and preparation process of Fast Gaussian Calculation (FGC), which is a fast and inaccurate dose calculation algorithm only performed in optimization stages, and leaf sequencing. FMO and SPO shared the same part of CPO process, which started with segment shape and weight optimizing to the end of dose calculation.

In the clinical operation of new systems and devices, necessary clinical validation is required. Existing studies^7^, ^8^, ^9^ have focused on dosimetry, planning complexity, and delivery efficiency, etc. However, the comprehensive study combining dosimetry, planning complexity, and execution efficiency and their relationship in this study is rare. The purpose of this study is to compare the FMO algorithm and SPO algorithm in terms of dosimetric parameters, plan complexity, delivery efficiency and their relationship for cervical cancer radiotherapy, to provide the basis for the algorithm selection of radiotherapy planning.

## 2 MATERIALS AND METHODS

### 2.1 Patient's clinical characteristics

We retrospectively reviewed 34 women who were diagnosed with stageIIA–IVB cervical cancer and treated with definitive radiation at the Sun Yat‐sen University Cancer Center from October 2021 to August 2022. There are 30 squamous cell carcinoma, 2 recurrent carcinoma, 1 adenocarcinoma and 1 small cell carcinoma of uterine cervix. The median age is 54.5 years (range from 42 to 75 years).

### 2.2 Simulation and contouring

Before simulation, patients were instructed to empty their bowel and fill the bladder. All patients were immobilized with a vacuum bag system with a supine position. Intravenous contrast‐enhanced CT simulation was done with a slice thickness of 3 mm for the whole abdomen and pelvic region on a Brilliance big bore (Philips, Amsterdam, Netherlands)) computed tomography (CT) simulator. The reconstructed CT images were transmitted to Monaco workstation (Version 5.11.01, Elekta, Sweden). The gross target volume (GTV) included all grossly enlarged lymph nodes with a diameter of ≥1 cm and regional metastatic lymph nodes on imaging findings. The clinical target volume (CTV) included the cervix, whole uterus, parametrium, upper part of the vagina, and pelvic lymphatic drainage area. In patients with common iliac matestatic lymph nodes, para‐aortic irradiation was administered. The planning target volume (PGTV/PCTV) was a uniform expansion of the GTV/CTV in the three‐dimensional direction by 5 mm.^10^ The OARs included the bladder, rectum, small intestine, colon, left/right femoral head, left/right kidney, spinal cord and pelvic bone.

### 2.3 Treatment planning

The prescribed dose 45 Gy was delivered to the PCTV with whole‐pelvis in 25 fractions, and a boost up to 60 Gy was delivered to PGTV. All plans were designed on UIH treatment planning system named uRT‐TPOIS (Version R001, United Imaging Healthcare Co., Shanghai, China) with 6 MV photon with uARC. Dose calculation was performed using Monte Carlo (MC) algorithm and was set with dose to medium. Two arcs of 360° was applied for uARC plans with gantry angles ranging from 0 to 360°. The statistical uncertainty of each plan was 1%, and the dose calculating grid size was 3 mm. The setting was applied in both optimization process and dose calculation. The optimization iteration was 50. The plans for the same patient were optimized in the same objectives and constraints as showed in Supplement Table S1. All the plans were optimized once. Four plans were made for each patients and the detailed setting was showed in Table 1.

**TABLE 1:** The optimization algorithm and jaw setting of the four plans.

	FMO‐FJ plans	FMO‐NFJ plans	SPO‐FJ plans	SPO‐NFJ plans
Optimization algorithm	FMO	FMO	SPO	SPO
Jaw setting (X:cm)	A1:X1/X2:−2/20; A2:X1/X2:−20/2;	A1:X1/X2:−20/20; A2:X1/X2:−20/20;	A1:X1/X2:−2/20; A2:X1/X2:−20/2;	A1:X1/X2:−20/20 A2:X1/X2:−20/20

*Note*: FMO‐FJ plans: FMO plans with fixed jaw; FMO‐NFJ plans: FMO plans with no fixed jaw.

SPO‐FJ plans: SPO plans with fixed jaw; SPO‐NFJ plans: SPO plans with no fixed jaw.

Y1/Y2 setting of the four plans were −20 cm/20 cm; A1/A2 refer to Arc1/Arc2.

### 2.4 Plan evaluation

Four cervical plans were compared in terms of target and OARs dosimetric indices, such as the homogeneity index (HI), conformity index (CI), maximum dose of target volume, target coverage (TC), MUs, control points, and the dose‐volume histogram (DVH) parameters concerning OARs, according to the International Commission on Radiation Units Report No. 83^11^ and the clinical constraints in our center. The HI and CI were calculated using formula (1) and (2), respectively:

(1) $$\mathrm{HI}=\frac{{}_{{}_{D_{2\%}}{-}_{D_{98\%}}}}{D_{50\%}}$$

(2) $$\mathrm{CI}=\frac{{}^{{\left(TV_{\mathrm{Target}100\%}\right)}^{2}}}{TV\times V_{\mathrm{Target}100\%}}$$

where D_2%_, D_98%_ and D_50%_ are the dose that cover 2%, 98% and 50% of target, respectively. The closer the CI value is to 1, the better the conformity of the target volume is. TV is the volume of the target; TV_Target100%_ is the target volume covered by the prescription dose, and V_Target100%_ represents the total volume covered by the prescription dose. The smaller the HI value, the more uniform the target dose.

The complexity metric is a useful tool for plan evaluation. All kinds of complexity metrics were studied for comparing different technology and TPS, or the validation of the new system.^12^, ^13^ Based on McNiven et al study,^14^ the modulation complexity score (MCS) combines two parameters: the leaf sequence variability (LSV) and the aperture area variability (AAV). The LSV parameter is used to characterize the variation in segment shape, and the AAV is used to characterize the variation in segment area relative to the maximum aperture defined by all the segments. The LSV and AAV for a given segment at a certain gantry angle are as described by Equations (4) and (5), respectively, where POS is the coordinate of the leaf position and N is the number of open leaves inside the jaw position. n is the total number of leaf pairs, and N is the index number of leaf pairs.

(3) $$\mathrm{PO}S_{\max}=\langle \max(\mathrm{po}s_{N\in n})-{\min(\mathrm{po}s_{N\in n})\rangle }_{\mathrm{leaf}\;\mathrm{bank}}$$

(4) $$\begin{array}{ccc} & & \mathrm{LS}V_{\mathrm{segment}}\\ & & ={\langle \frac{\sum_{n=1}^{N}(\mathrm{po}s_{\max}-\left(pos_{n}-pos_{n+1}\right))}{N\times pos_{\max}}\rangle }_{\mathrm{left}\;\mathrm{bank}}\\ & & \times \;{\langle \frac{\sum_{n=1}^{N}(pos_{\max}-(pos_{\max}-\left(pos_{n}-pos_{n+1}\right)}{N\times pos_{\max}}\rangle }_{\mathrm{right}\;\mathrm{bank}} \end{array}$$

(5) $$\begin{array}{ccc} & & \mathrm{AA}V_{\mathrm{segment}}\\ & & =\frac{\sum_{n=1}^{N}{\langle pos_{n}\rangle }_{\mathrm{left}\;\mathrm{bank}}-{\langle \mathrm{po}s_{n}\rangle }_{\mathrm{right}\;\mathrm{bank}}}{\sum_{n=1}^{N}{\langle \max(\mathrm{po}s_{n})\rangle }_{\mathrm{left}\;\mathrm{bank}\;\in \;\mathrm{arc}}-{\langle \max(\mathrm{po}s_{n})\rangle }_{\mathrm{right}\;\mathrm{bank}\;\in \;\mathrm{arc}}} \end{array}$$

To summarize the influence of the LSV, AAV and MU, the MCS for a given arc is calculated based on the index above, as described in Equation (6):

(6) $$MCS_{\mathrm{arc}}=\sum_{i=1}^{I}AAV_{\mathrm{segment}\;i}\times LSV_{\mathrm{segement}\;i}\times \frac{MU_{\mathrm{segment}\;i}}{MU_{\mathrm{arc}}}$$

Where *I* is the number of the segments in the arc.

The total plan complexity is defined by the MCS_plan_ metric. The MCS_plan_ is the MCS_arc_ weighted by the relative MU of each arc in the plan, as described in Equation (7):

(7) $$MCS_{\mathrm{plan}}=\sum_{j=1}^{J}MCS_{\mathrm{arc}\;j}\times \frac{MU_{\mathrm{arc}\;j}}{MU_{\mathrm{plan}}}$$

where *J* is the number of arcs in the total plan.

MCSs ranged from 0 to 1. The lower the value of the MCS is, the higher the plan complexity is.

The MapCheck3 (SNC, USA) two‐dimensional diode matrix was used to verify the dose for all the plans in our study. All measurements were performed with the actual beam angle. The dose plane calculated by the TPS was compared with the measured dose of the MapCheck3 to calculate the global absolute dose gamma (γ) value (use the maximum dose as the denominator to calculate the dose difference) and the gamma pass rate (GPR) of the plans. To eliminate the effects of low‐dose signals, the lower limit of the dose threshold in γ calculations is set at 10%. The global γ indices are considered clinically acceptable if the GPR (3%/3 mm) is ≥95%.

### 2.5 Statistical analysis

Pairwise comparisons of the targets and OARs dose in the four plans were performed using SPSS version 22.0 (IBM Corp., Armonk, NY). Paired‐T test was used when the data was normally distributed, otherwise the Wilcoxon test was applied. Analysis items with *P* < 0.05 were considered statistically significant. The correlation of the plan MCS, MUs, delivery time, GPR and PCTV width/length was investigated using the Spearman's correlation coefficients, r_s_. The strength of the correlations was evaluated with a significance level of 5%.

## 3 RESULTS

### 3.1 Difference of the FMO‐FJ, FMO‐NFJ, SPO‐FJ, SPO‐NFJ plans

#### 3.1.1 Targets coverage and OARs sparing

**FIGURE 2:**
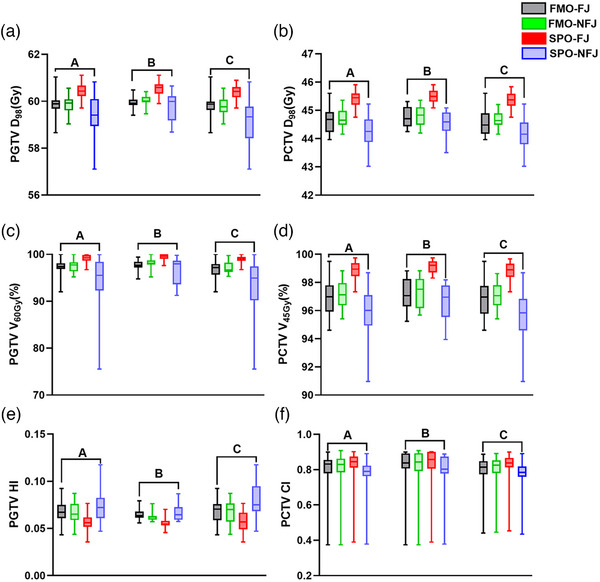
Comparison of target coverage of the four plans in patients (**Group a**: represents target difference of the 34 patients; **Group b**: represents target difference of the patients with PCTV width ≤15.5 cm; **Group c**: represents target difference of the patients with PCTV width >15.5 cm).

The target dose of the four uARC plans are shown in Table 2. For the four plans, the PGTV V66Gy is 0% for all plans and there is no difference between these plans. Moreover, SPO‐FJ plans are the best plans, because it significantly outperformed the other plans in terms of PGTV (D98%, V60Gy, HI) and PCTV (D98%, V45Gy, CI) (*P* ≤ 0.00). FMO plans are better than SPO‐NFJ plans (*P* ≤ 0.00). In other words, SPO‐NFJ plans are the worst ones. No significant difference is found between FMO‐FJ and FMO‐NFJ plans (*P* ≥ 0.08). Details are showed in **Group a** in Figure 2 and Table 2.

**TABLE 2:** Target coverage of four plans (FMO‐FJ, FMO‐NFJ, SPO‐FJ, SPO‐NFJ) for 34 cervical cancer patients.

	FMO‐FJ	FMO‐NFJ	SPO‐FJ	SPO‐NFJ	*P1*	*P2*	*P3*	*P4*	*P5*	*P6*
PGTV										
D_98%_(Gy)	59.87 ± 0.43	59.88 ± 0.38	60.44 ± 0.31	59.39 ± 0.84	0.00	0.00	0.00	0.81	0.00	0.00
V_66Gy_(%)	0.00 ± 0.00	0.00 ± 0.00	0.00 ± 0.00	0.00 ± 0.00	0.18	0.33	0.25	0.66	0.16	0.40
V_60Gy_(%)	0.97 ± 0.02	0.97 ± 0.01	0.99 ± 0.01	0.94 ± 0.06	0.00	0.00	0.00	0.31	0.00	0.00
CI	0.60 ± 0.14	0.60 ± 0.14	0.58 ± 0.13	0.54 ± 0.15	0.00	0.00	0.01	0.21	0.00	0.00
HI	0.07 ± 0.01	0.07 ± 0.01	0.06 ± 0.01	0.07 ± 0.02	0.00	0.00	0.00	0.46	0.00	0.00
PCTV										
D_98%_(Gy)	44.62 ± 0.42	44.70 ± 0.32	45.4 ± 0.27	44.27 ± 0.55	0.00	0.00	0.00	0.14	0.00	0.00
V_45Gy_(%)	0.97 ± 0.01	0.97 ± 0.01	0.99 ± 0.01	0.96 ± 0.02	0.00	0.00	0.00	0.08	0.00	0.00
CI	0.79 ± 0.11	0.8 ± 0.12	0.82 ± 0.11	0.77 ± 0.11	0.00	0.00	0.00	0.27	0.00	0.00

*Note*: *P1*: FMO‐NFJ *vs* SPO‐NFJ; *P*2: SPO‐FJ *vs* SPO‐NFJ; *P*3: SPO‐FJ *vs* FMO‐FJ; *P*4: FMO‐NFJ *vs* FMO‐FJ.

*P*5: SPO‐NFJ *vs* FMO‐FJ; *P*6: FMO‐NFJ *vs* SPO‐FJ.

*P* < 0.05 indicates significant difference; Values are showed as mean ± SD.

**FIGURE 3:**
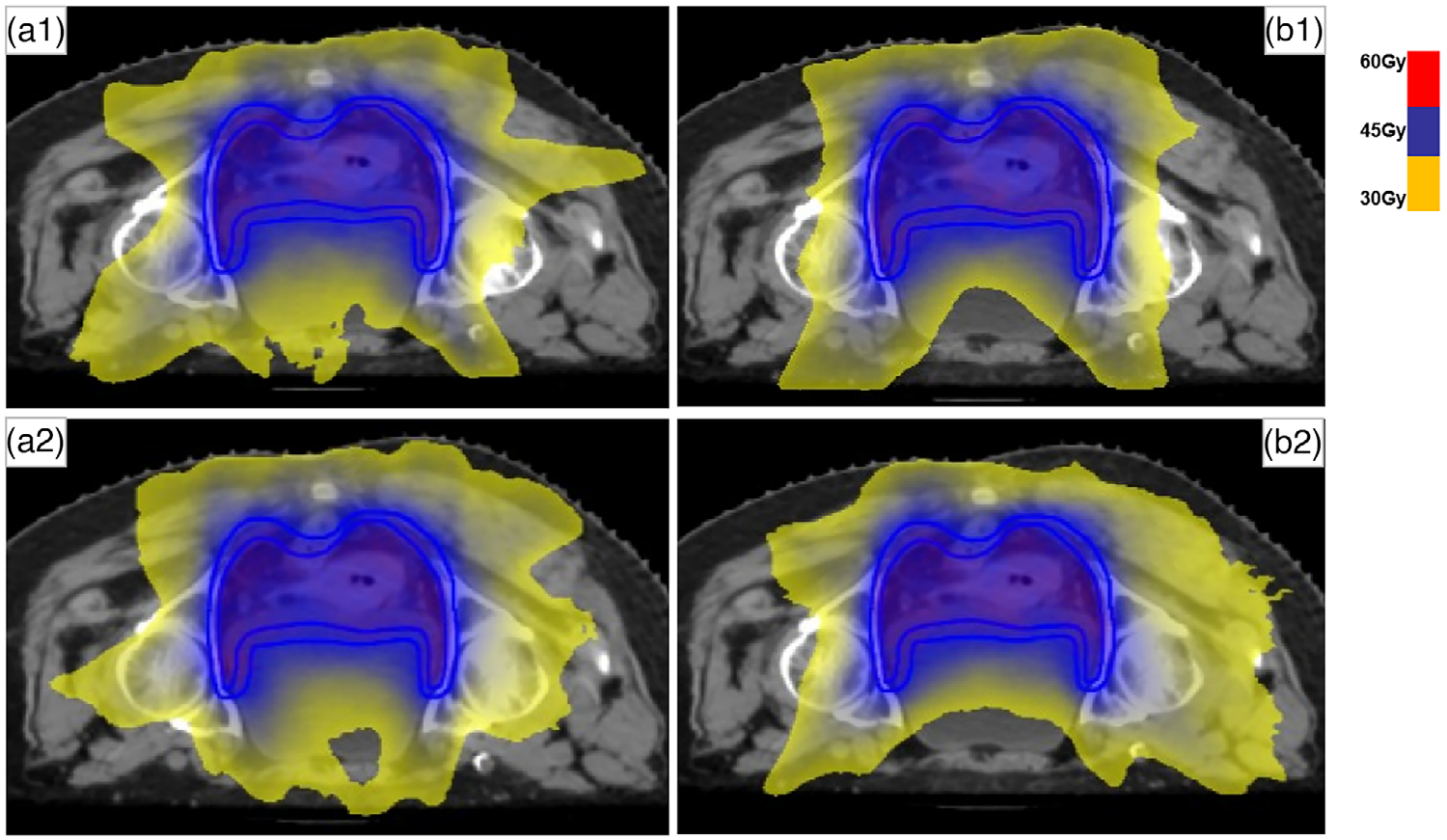
Dose distribution display for the four plans (a1: FMO‐FJ plan; a2: FMO‐NFJ plan; b1: SPO‐FJ plan; b2: SPO‐NFJ plan).

For OARs sparing, most organs in SPO‐FJ plans has more superiority than those in FMO plans (*P* ≤ 0.04), except for kidney L/R (Dmean,V18Gy), rectum V45Gy (*P* ≥ 0.13). Additionally, OARs in SPO‐FJ plans has lower dose than those in SPO‐NFJ plans (*P* ≤ 0.02), except for rectum V45Gy and kidney‐R Dmean (*P* ≥ 0.12). Compared with SPO‐NFJ plans, FMO plans delivered lower dose to bladder (Dmean,V40Gy), rectum (Dmean, V40Gy, V45Gy), Kidney‐R V18Gy, Colon V40Gy, and Pelvic bone V40Gy (*P* ≤ 0.04). Furthermore, FMO‐FJ plans showed minor superiority than FMO‐NFJ plans in kidney L/R Dmean and small intestine V30Gy (*P* ≤ 0.04), and no difference was found for the others, Details can be found in Table 3 and Supplement Figure S1. The dose distribution difference was displayed in Figure 3.

**TABLE 3:** Dosimetric difference of OARs in the four plans.

	FMO‐FJ	FMO‐NFJ	SPO‐FJ	SPO‐NFJ	*P1*	*P2*	*P3*	*P4*	*P5*	*P6*
**Bladder**										
D_mean_ (Gy)	40.43 ± 2.51	40.3 ± 2.65	39.08 ± 3.3	41.37 ± 3.3	0.00	0.00	0.00	0.21	0.00	0.00
V_30Gy_(%)	0.88 ± 0.10	0.87 ± 0.11	0.80 ± 0.13	0.89 ± 0.11	0.09	0.00	0.00	0.41	0.22	0.00
V_40Gy_(%)	0.58 ± 0.12	0.57 ± 0.12	0.54 ± 0.12	0.62 ± 0.15	0.00	0.00	0.00	0.34	0.001	0.00
**Rectum**										
D_mean_(Gy)	43.48 ± 1.49	43.42 ± 1.53	43.22 ± 1.63	43.82 ± 1.84	0.00	0.00	0.00	0.19	0.00	0.04
V_40Gy_(%)	0.8 ± 0.08	0.8 ± 0.09	0.78 ± 0.09	0.81 ± 0.09	0.00	0.00	0.00	0.39	0.02	0.00
V_45Gy_(%)	0.55 ± 0.09	0.54 ± 0.1	0.56 ± 0.09	0.57 ± 0.10	0.00	0.54	0.19	0.14	0.04	0.04
**Kidney**										
L‐D_mean_(Gy)	10.07 ± 5.57	10.35 ± 5.68	10.00 ± 5.53	10.2 ± 5.53	0.12	0.00	0.13	0.00	0.04	0.00
L‐V_18Gy_(%)	0.14 ± 0.1	0.15 ± 0.1	0.14 ± 0.09	0.15 ± 0.09	0.30	0.00	0.31	0.51	0.04	0.28
R‐D_mean_(Gy)	10.43 ± 5.47	10.59 ± 5.5	10.48 ± 5.49	10.61 ± 5.53	0.81	0.12	0.29	0.01	0.08	0.20
R‐V_18Gy_(%)	0.14 ± 0.08	0.14 ± 0.08	0.14 ± 0.08	0.15 ± 0.08	0.00	0.00	0.86	0.76	0.00	0.85
**Spinal cord**										
D_max_(Gy)	34.3 ± 2.50	34.4 ± 2.45	32.66 ± 3.42	35.11 ± 3.81	0.10	0.00	0.00	0.44	0.06	0.00
**Femoral Head**										
L‐V_30Gy_(%)	0.12 ± 0.08	0.12 ± 0.07	0.08 ± 0.05	0.13 ± 0.07	0.49	0.00	0.00	0.98	0.51	0.00
R‐V_30Gy_(%)	0.11 ± 0.09	0.13 ± 0.08	0.09 ± 0.05	0.12 ± 0.07	0.90	0.00	0.01	0.07	0.14	0.00
**Small intestine**										
V_40Gy_(cc)	79.58 ± 51.21	80.59 ± 52.84	76.18 ± 48.75	80.79 ± 51.97	0.84	0.02	0.02	0.21	0.36	0.01
V_30Gy_(cc)	211.22 ± 115.44	217.16 ± 119.12	194.3 ± 106.63	220.43 ± 122.13	0.46	0.00	0.00	0.04	0.06	0.00
**Colon**										
V_40Gy_(cc)	60.93 ± 37.09	61.63 ± 38.46	58.74 ± 35.61	64.44 ± 40.51	0.00	0.00	0.00	0.11	0.00	0.01
V_30Gy_(cc)	95.09 ± 55.06	96.87 ± 57.20	90.53 ± 51.30	95.84 ± 54.50	0.52	0.00	0.00	0.16	0.61	0.01
**Pelvic bone**										
D_mean_(Gy)	29.78 ± 1.68	29.86 ± 1.70	29.07 ± 1.65	29.89 ± 1.67	0.77	0.00	0.00	0.21	0.21	0.00
V_40Gy_(%)	0.26 ± 0.04	0.26 ± 0.04	0.25 ± 0.03	0.27 ± 0.04	0.01	0.00	0.00	0.44	0.00	0.00
V_30Gy_(%)	0.55 ± 0.05	0.56 ± 0.05	0.52 ± 0.04	0.55 ± 0.04	0.08	0.00	0.00	0.46	0.17	0.00
V_20Gy_(%)	0.73 ± 0.05	0.73 ± 0.05	0.71 ± 0.05	0.73 ± 0.05	0.59	0.00	0.00	0.61	0.83	0.00
V_10Gy_(%)	0.87 ± 0.08	0.87 ± 0.08	0.86 ± 0.09	0.87 ± 0.08	0.32	0.00	0.00	0.24	0.93	0.00

*Note*: *P1*: FMO‐NFJ *vs* SPO‐NFJ; *P*2: SPO‐FJ *vs* SPO‐NFJ; *P*3: SPO‐FJ *vs* FMO‐FJ; *P*4: FMO‐NFJ *vs* FMO‐FJ; *P*5: SPO‐NFJ *vs* FMO‐FJ; *P*6: FMO‐NFJ *vs* SPO‐FJ. “L/R” represents “Left/Right”; *P* < 0.05 indicates significant difference.

#### 3.1.2 MCS, MUs, delivery time and GPR

Table 4 presents the comparison of MCSs, MU, delivery time and GPR of the four uARC plans. MUs of SPO‐NFJ plans are significantly smaller than the other plans (*P* ≤ 0.00). Moreover, MUs of SPO‐FJ plans are lower than FMO‐FJ plans (*P* = 0.04), and have no difference with FMO‐NFJ plans.

**TABLE 4:** Comparison of MCS, Mu, delivery and GPR of the four plans.

	FMO‐FJ	FMO‐NFJ	SPO‐FJ	SPO‐NFJ	*P1*	*P2*	*P3*	*P4*	*P5*	*P6*
Time(s)	165.12 ± 12.06	170.17 ± 5.78	126.74 ± 4.78	123.95 ± 4.73	0.00	0.00	0.00	0.01	0.00	0.00
MCS	0.18 ± 0.03	0.17 ± 0.03	0.19 ± 0.03	0.23 ± 0.05	0.00	0.00	0.01	0.14	0.00	0.00
Mu	853.82 ± 120.13	790 ± 99.91	793.2 ± 95.37	535.12 ± 104.9	0.00	0.00	0.04	0.02	0.00	0.88
GPR(%)	96.04 ± 1.84	96.4 ± 1.84	95.46 ± 2.03	97.21 ± 1.53	0.01	0.00	0.13	0.12	0.00	0.04

*Note*: *P1*: FMO‐NFJ *vs* SPO‐NFJ; *P*2:SPO‐FJ *vs* SPO‐NFJ; *P*3: SPO‐FJ *vs* FMO‐FJ; *P*4: FMO‐NFJ *vs* FMO‐FJ.

*P*5: SPO‐NFJ *vs* FMO‐FJ; *P*6: FMO‐NFJ *vs* SPO‐FJ. *P* < 0.05 indicates significant difference.

The delivery time of SPO plans is much lower than FMO plans (*P* = 0.00), and that of SPO‐FJ plans are slightly more than SPO‐NFJ plans. The time of FMO‐FJ is lower than FMO‐NFJ plans (*P* = 0.01).

The MCS of SPO plans are higher than FMO plans (*P* ≤ 0.01), and that of SPO‐NFJ plans are higher than SPO‐FJ plans (*P* = 0.00). The MCS of SPO‐NFJ plans are significantly highest in the four plans (*P* = 0.00). No significant difference is found between FMO‐FJ plans and FMO‐NFJ plans.

In addition, the GPR of SPO‐NFJ plans are the highest in the four plans (*P* ≤ 0.01). The GPRs of SPO‐FJ plans are slightly lower than that of FMO‐NFJ plans (*P* = 0.04), and have no difference with FMO‐FJ plans.

In all, SPO‐NFJ plans show superiority in MU, delivery time, MCS and GPR among the four plans. In terms of delivery time, MU and MCS, SPO‐FJ plans are better than FMO plans. The FMO‐FJ plans are better than FMO‐NFJ plans in delivery time.

We analyzed the correlation of MCSs, MUs, delivery time, GPRs and PCTV width/length. As Table 5 reveals, MCSs are strongly correlated with PCTV length, except for SPO‐NFJ plans. The GPR of the four plans showed a negative correlation with PCTV length (*P* ≤ 0.03). MUs of all the plans have no correlation with PCTV width and length. In addition, delivery time of the plans is not correlated with PCTV length. In addition, MUs of SPO plans are negatively correlated with MCSs (*P* ≤ 0.02). In addition, the delivery time and MUs of the four plans are strongly correlated (*P* ≤ 0.02). Details can be found in Supplement Figure S2.

**TABLE 5:** Spearman's Correlation of PCTV width and length with MCS, MU, GPR and delivery time of the four plans.

	MU				MCSs				GPR				Delivery Time			
	FMO‐NFJ	FMO‐FJ	SPO‐NFJ	SPO‐FJ	FMO‐NFJ	FMO‐FJ	SPO‐NFJ	SPO‐FJ	FMO‐NFJ	FMO‐FJ	SPO‐NFJ	SPO‐FJ	FMO‐NFJ	FMO‐FJ	SPO‐NFJ	SPO‐FJ
Width	0.291 (0.09)	0.09 (0.61)	0.11 (0.53)	0.21 (0.24)	0.33 (0.06)	**0.36** **(0.04)**	0.15 (0.41)	0.26 (0.14)	−0.21 (0.29)	−0.28 (0.16)	−0.22 (0.28)	−0.11 (0.59)	0.28 (0.11)	**0.48** **(0.00)**	**0.48** **(0.00)**	0.15 (0.39)
Length	0.01 (0.94)	−0.22 (0.20)	0.15 (0.40)	0.11 (0.56)	**0.56** **(0.00)**	**0.75** **(0.00)**	0.16 (0.37)	**0.53** **(0.00)**	**‐0.71** **(0.00)**	**‐0.76** **(0.00)**	**‐0.59** **(0.00)**	**‐0.41** **(0.03)**	0.14 (0.43)	0.15 (0.40)	0.27 (0.13)	−0.05 (0.79)

*Note*: The correlations was described in correlation coefficients (corresponding *p*) (PCTV width was the biggest distance in left‐right direction when gantry is 0°, and the length was the length of PCTV in superior‐inferior direction).

### 3.2 Difference of the FMO‐FJ, FMO‐NFJ, SPO‐FJ, SPO‐NFJ plans with different PCTV width

#### 3.2.1 Targets coverage and OARs sparing with different PCTV width

Thirt‐four patients’ plans were divided to two group with PCTV width of 15.5 cm as the cut‐off point. One group is 11 patients with PCTV width ≤15.5 cm, and the other group is 23 patients with PCTV width >15.5 cm. The target dose difference of the two groups is shown in Table 6. For target dose, no difference was found in FMO‐FJ and FO‐NFJ plans in the two groups. For PCTV width ≤15.5 cm, SPO‐FJ plans are better than SPO‐NFJ plans (*P <* 0.05), except for PGTV CI. For PCTV width >15.5 cm group, SPO‐FJ plans are significantly different from SPO‐NFJ, except for PGTV V66Gy. Details are showed as **Group b and c in** Figure 2.

**TABLE 6:** Target difference between fixed jaw plans and no fixed jaw plans with different PCTV width (15.5 cm as the cutoff).

	≤15.5 cm		>15.5 cm		≤15.5 cm		>15.5 cm	
PCTV Width	FMO‐FJ	FMO‐NFJ	FMO‐FJ	FMO‐NFJ	SPO‐FJ	SPO‐NFJ	SPO‐FJ	SPO‐NFJ
PGTV D_98%_(Gy)	59.95 ± 0.27	60.02 ± 0.27	59.83 ± 0.49	59.82 ± 0.41	60.52 ± 0.33^a^	59.77 ± 0.65	60.4 ± 0.31^a^	59.21 ± 0.87
PGTV V_66Gy_(%)	0.00 ± 0.00	0.00 ± 0.00	0.00 ± 0.00	0.00 ± 0.00	0.00 ± 0.00^a^	0.00 ± 0.00	0.00 ± 0.00	0.00 ± 0.00
PGTV V_60Gy_(%)	0.98 ± 0.01	0.98 ± 0.01	0.97 ± 0.02	0.97 ± 0.01	0.99 ± 0.01^a^	0.97 ± 0.03	0.99 ± 0.01^a^	0.93 ± 0.06
PGTV CI	0.6 ± 0.18	0.6 ± 0.18	0.6 ± 0.11	0.6 ± 0.12	0.57 ± 0.18	0.56 ± 0.16	0.58 ± 0.11^a^	0.53 ± 0.14
PGTV HI	0.06 ± 0.01	0.06 ± 0.01	0.07 ± 0.01	0.07 ± 0.01	0.06 ± 0.01^a^	0.07 ± 0.01	0.06 ± 0.01^a^	0.08 ± 0.02
PCTV D_98%_(Gy)	44.76 ± 0.35	44.8 ± 0.36	44.55 ± 0.44	44.65 ± 0.3	45.48 ± 0.24^a^	44.53 ± 0.46	45.36 ± 0.28^a^	44.14 ± 0.55
PCTV V_45Gy_(%)	0.97 ± 0.01	0.97 ± 0.01	0.97 ± 0.01	0.97 ± 0.01	0.99 ± 0.00^a^	0.97 ± 0.01	0.99 ± 0.01^a^	0.96 ± 0.02
PCTV CI	0.79 ± 0.16	0.79 ± 0.16	0.79 ± 0.09	0.80 ± 0.09	0.80 ± 0.16^a^	0.77 ± 0.15	0.82 ± 0.09^a^	0.77 ± 0.09

^a^ represents difference with SPO‐NFJ (*P* < 0.05).

**FIGURE 4:**
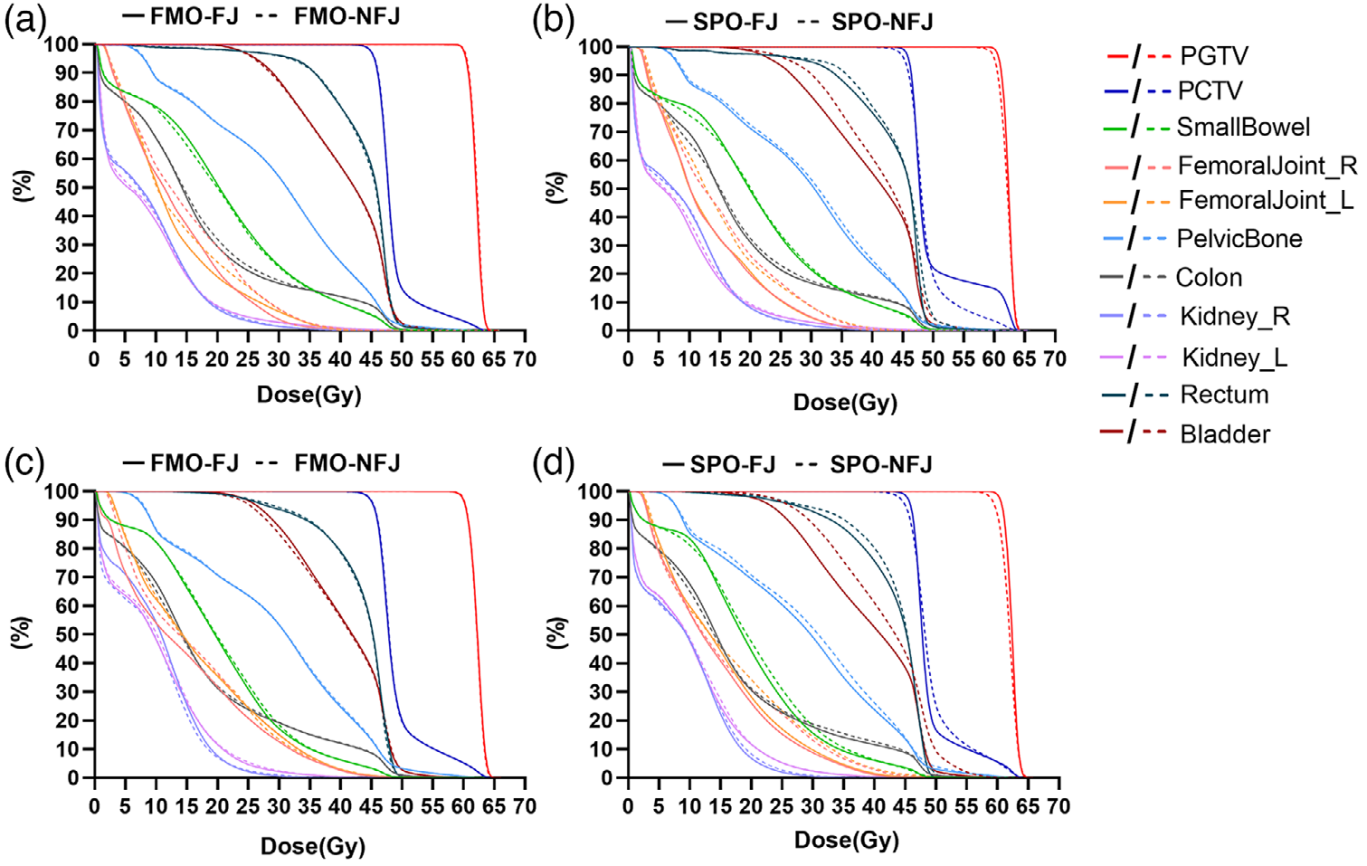
Averaged DVH difference of plans with PCTV width ≤15.5 cm (a,b) and PCTV width >15.5 cm (c,d).

For OARs sparing, For PCTV width ≤15.5 cm, only Kidney_L Dmean reveals minor difference between FMO‐FJ and FMO‐NFJ plans (*P* < 0.05). Most OARs of SPO‐FJ plans got lower dose than SPO‐NFJ plans, except for rectum V45Gy, Kidney‐R (Dmean, V18Gy), Femoral Head R V30Gy, Small intestine V40Gy and colon (V30Gy, V40Gy). For PCTV width >15.5 cm, Kidney L/R Dmean, Small intestine V30Gy in FMO‐FJ plans showed slightly lower dose than FMO‐NFJ (*P* < 0.05). In addition, most OARS dose of SPO‐FJ plans is significantly lower than those in SPO‐NFJ plans, except for rectum V45Gy, Kidney‐R Dmean. Details can be found in Figure 4 and Supplement Table S2.

#### 3.2.2 MCSs, MUs, delivery time and GPR with different PCTV width

For PCTV width ≤15.5 cm, MUs of FMO‐FJ plans is more than FMO‐NFJ plans, and delivery time is lower than FMO‐NFJ plans (*P* < 0.05). Moreover, MU, MCS, GPR and delivery time of SPO‐FJ plans are significantly different from SPO‐NFJ. For PCTV width >15.5 cm, MU, MCS, GPR and delivery time of SPO‐FJ plans are significantly different from SPO‐NFJ plans. No significant difference was found between FMO‐FJ and FMO‐NFJ plans (Table 7).

**TABLE 7:** MCSs, Mu, delivery and GPR difference between fixed jaw plans and no fixed jaw plans.

Width	≤15.5 cm		>15.5 cm		≤15.5 cm		>15.5 cm	
	FMO‐FJ	FMO‐NFJ	FMO‐FJ	FMO‐NFJ	SPO‐FJ	SPO‐NFJ	SPO‐FJ	SPO‐NFJ
MU	852.66 ± 99.51	742.36 ± 122.99^a^	854.37 ± 130.93	812.79 ± 80.11	766.88 ± 60.74	527.66 ± 87.2^b^	805.79 ± 107.02	538.68 ± 114.06^b^
MCS	0.16 ± 0.02	0.16 ± 0.03	0.18 ± 0.03	0.18 ± 0.02	0.17 ± 0.02	0.21 ± 0.04^b^	0.19 ± 0.03	0.24 ± 0.05^b^
GPR(%)	96.49 ± 1.82	96.77 ± 1.89	95.74 ± 1.84	96.14 ± 1.81	95.24 ± 2.48	97.35 ± 1.45^b^	95.61 ± 1.72	97.11 ± 1.63^b^
Time(S)	161.81 ± 8.36	168.42 ± 6.18^a^	166.7 ± 13.35	171.01 ± 5.52	125.7 ± 3.03	121.23 ± 2.00^b^	127.24 ± 5.41	125.25 ± 5.13^b^

^a^ represents significant difference with FMO‐FJ plans (*P* < 0.05).

^b^ represents significant difference with SPO‐FJ plans (*P* < 0.05).

## 4 DISSCUSSION

Plan dosimetry quality and delivery efficiency are two aspects of compromise that higher delivery gantry speed leads to a lower dosimetry quality under normal conditions. For conventional fractionated radiotherapy, the maximum moving distance of all MLC leaves between two adjacent control points is the main factor affecting the plan quality and speed. Longer moving distance of leaves means a higher modulation ability but a slower delivery speed for the gantry speed should be reduced to wait for the leaves moving to the positions.

VMAT plans for cervical cancer patients produce a large number of long, small, and irregular segments. It would result in complicated planning and long treatment time. Currently, many studies have focused on minimizing the delivery time while ensuring the quality of radiotherapy planning.^15^, ^16^, ^17^, ^18^ With the introduction of UIH 506C linac and TPS, we investigated the four plans difference in the two optimization algorithm with different fixed jaw to provide a basis for the selection of clinical radiotherapy techniques.

Target coverage and OARs sparing are crucial to the tumor control and radiation toxicity.^10^ Our results showed that SPO‐FJ plans could provide best target coverage and least dose to OARs in the four plans. In contrast, SPO‐NFJ plans are the worst in the four plans. There was no significant difference between FMO‐FJ plans and FMO‐NFJ plans. Further experimental results showed that: The FMO‐FJ, FMO‐NFJ and SPO‐NFJ need to be re‐optimized 3, 3 and 6 times to achieve the similar dose distribution of SPO‐FJ plans. The once optimization of SPO‐FJ plans could reduce optimizing time and the dependence of the planners. In terms of the dose distribution and optimizing efficiency, the SPO‐FJ algorithm was the best choice for the cervical cancer radiotherapy.

The SPO‐FJ plans are comparable to SPO‐NFJ plans in delivery time, which is at least 38s less than FMO plans on average. The MCS incorporates the leaf sequence variability and the aperture area variability into the calculation. In terms of delivery time, and MCS, SPO‐FJ plans are better than FMO plans. FMO‐FJ plans are better than FMO‐NFJ plans in delivery time. All treatment plans showed good GPRs; the mean GPR (3%/3 mm) was >95%. Increased delivery efficiency could reduce intra‐fraction motion, and also increase patient comfort.^19^, ^20^ Less beam modulation gives less head leakage and less collimator scattering, which could have an impact on the effort to decrease out‐of‐field dose and secondary cancers.^21^ In a word, SPO‐FJ plans would be the best choice for cervical cancer radiotherapy.

**FIGURE 5:**
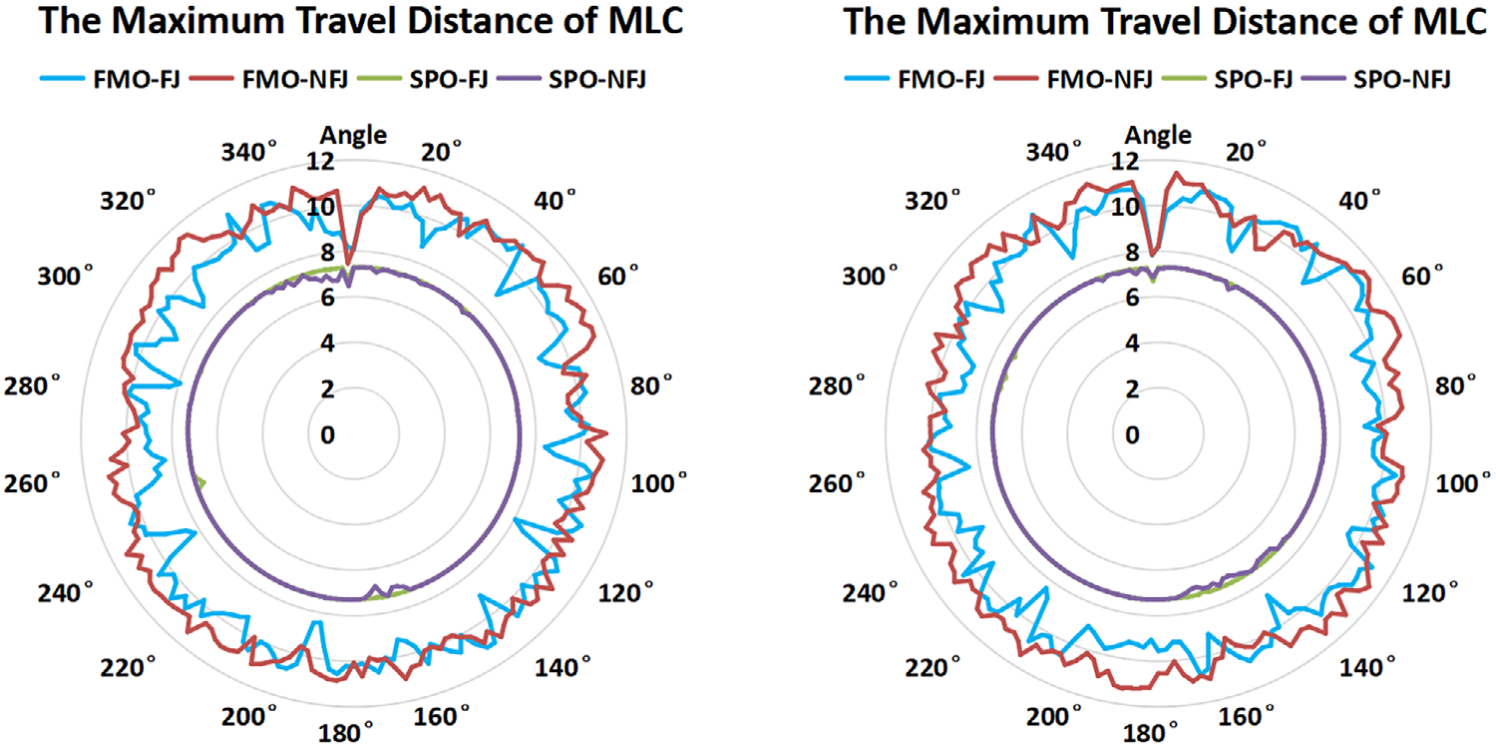
The averaged maximum travel distance of MLC (Left: beam Arc 1; Right: beam Arc 2; Rings represent different distances in mm.).

Studies on correlation of MCS, GRP, Mu and targets size showed diversity.^12^, ^22^, ^23^ In our study, MCS showed negative correlation with MUs, and it means more plan modulation contribute to more MUs. As results that MCS is not predicating factor for plan pass rate,^12^, ^24^ our studies showed no correlation between MCS and GPR. In addition, MCS was strongly correlated with PCTV length and not correlated with delivery time. This may be related to the optimization algorithm setting, such as that SPO plans were optimized with gantry maximum speed. Moreover, our results showed that SPO plans take less delivery time than FMO plans, it may contribute to the shorter travel distance of MLC, as displayed in Figure 5.

Researchers found that appropriate field fixed jaw setting showed superiority in OARs sparing.^6^, ^25^, ^26^, ^27^ In our studies, SPO‐FJ (fixed jaw) plans were significantly better than SPO‐NFJ (no fixed jaw) plans both in target coverage and OARs sparing, no matter what the width of PCTV is. However, for FMO plans, there was no significant difference between FMO‐FJ plans and FMO‐NFJ plans. These contributed to the adaptively sub‐regional irradiation according to the target size in FMO algorithm setting. It resulted in similar MCS, GPR, and minor difference (6.39s) in delivery time between FMO‐FJ and FMO‐NFJ plans, as results in Tables 4 and 7. Moreover, this can explain why there is no difference in target coverage and mostly OARs for two FMO plans (in Table 6), no matter what the width of the PCTV is. However, it is different for SPO algorithm setting. When the jaw of SPO plans was not fixed, MLC would travel in the whole target, MCS would increase and MU decline, the modulation ability of the plan was poor, resulting in poor dose distribution. This was the reason for the worst performance in dose mapping and biggest MCSs among the four plans. In contrast, for SPO plans with fixed jaw, as the modulation capacity increases, the dose mapping gets better. That is why SPO‐FJ plans was best in target coverage and OARs sparing in the four plans. Although the MUs increased, the delivery time was comparable to SPO‐NFJ plans and lower than FMO plans because of the setting of the maximum gantry speed. All in all, the best dose mapping and better delivery efficiency reveals the best performance of SPO‐FJ plans for cervical cancer radiotherapy.

Our study explored the difference of different algorithm and jaw fixing in cervical cancer radiotherapy. There are some limits in our study: (1) our study is limited to cervical cancer, and we would investigate in more site/disease in the future. (2) The jaw setting in our study is in only one way, more different setting should be explored. All these would be done in our future study.

## 5 CONCLUSION

In summary, for the newly‐introduced TPS, the GPRs of FMO plans and SPO plans can meet clinical requirements. For plans of cervical cancer radiotherapy, SPO optimization algorithm with fixed jaw can obtain the best target dose mapping and least dose to OARs.

## AUTHOR CONTRIBUTIONS

Sijuan Huang designed the whole experiment and wrote the manuscript. Jinlong Du and Yujie Du designed the plans in the study. Xin Yang and Xiaoyan Huang supervised the whole study. Xiuying Mai and Xi Lin collected and analyzed the data. Hongdong Liu, Wenzhao Sun, Kang Zhang and Jinhan Zhu provided technical assistance for the study. All co‐authors have reviewed and approved the final manuscript.

## CONFLICT OF INTEREST STATEMENT

The authors have no conflict of interest to declare in relation to this study.

## Supporting information

Supporting Information

## Data Availability

The datasets are fully available up on the Research Data Deposit platform (RDD Number:RDDA2022843350, https://www.researchdata.org.cn) and are available upon reasonable request.
