# Supplementary material for: Plan quality and treatment efficiency assurance of two VMAT optimization for cervical cancer radiotherapy
Source: J Appl Clin Med Phys. 2023 May 29;24(10):e14050. doi: 10.1002/acm2.14050 (PMC10562038; doi:10.1002/acm2.14050)
Supplement: Supplementary file 1 — Supporting Information [file ACM2-24-e14050-s001.docx]

**Supplement:**

**
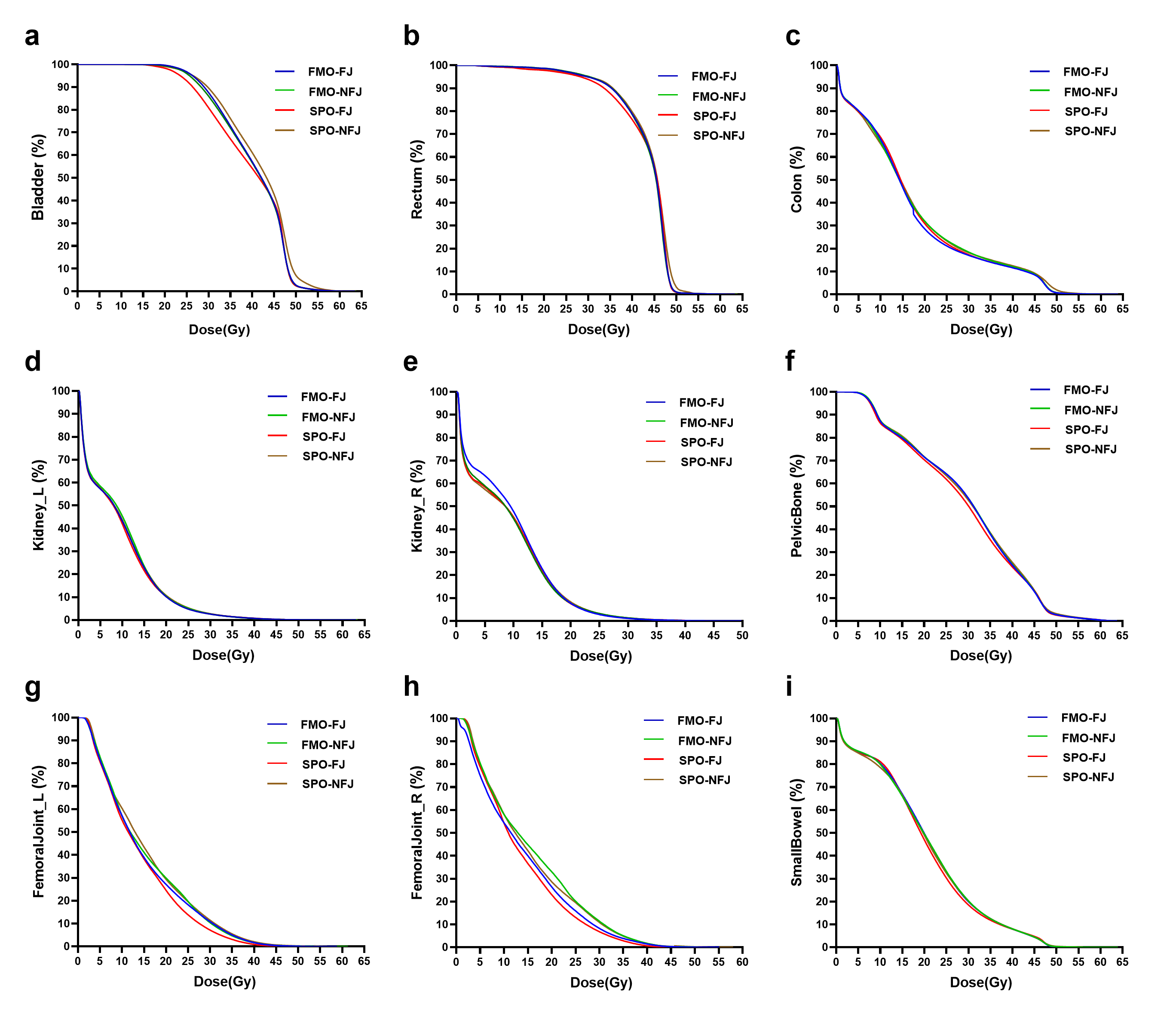
**

**FIGURE S1**  Averaged DVH difference of OARs in the four plans

| Cor |  | MCS | | | | GPR | | | | Delivery Time | | | |
| --- | --- | --- | --- | --- | --- | --- | --- | --- | --- | --- | --- | --- | --- |
|  |  | FMO-NFJ | FMO-FJ | SPO-NFJ | SPO-FJ | FMO-NFJ | FMO-FJ | SPO-NFJ | SPO-FJ | FMO-NFJ | FMO-FJ | SPO-NFJ | SPO-  FJ |
| MU | FMO-NFJ | -0.26  (0.14) |  |  |  | -0.10  (0.62) |  |  |  | **0.39**  **(0.02)** |  |  |  |
|  | FMO-FJ |  | -0.30  (0.08) |  |  |  | 0.20  (0.32) |  |  |  | **0.39**  **(0.02)** |  |  |
|  | SPO-NFJ |  |  | **-0.82**  **(0.00)** |  |  |  | -0.28  (0.16) |  |  |  | **0.55**  **(0.00)** |  |
|  | SPO-FJ |  |  |  | **-0.39**  **(0.02)** |  |  |  | -0.35  (0.07) |  |  |  | **0.64**  **(0.00)** |
| MCS | FMO-NFJ | 1 |  |  |  | -0.20  (0.32) |  |  |  | 0.08  (0.64) |  |  |  |
|  | FMO-FJ |  | 1 |  |  |  | **-0.47**  **(0.01)** |  |  |  | 0.11  (0.53) |  |  |
|  | SPO-NFJ |  |  | 1 |  |  |  | 0.26  (0.18) |  |  |  | -0.25  (0.15) |  |
|  | SPO-FJ |  |  |  | 1 |  |  |  | 0.05  (0.80) |  |  |  | **-0.40**  **(0.02)** |
| GPR | FMO-NFJ |  |  |  |  | 1 |  |  |  | 0.07  (0.74) |  |  |  |
|  | FMO-FJ |  |  |  |  |  | 1 |  |  |  | -0.39  (0.05) |  |  |
|  | SPO-NFJ |  |  |  |  |  |  | 1 |  |  |  | -0.22  (0.28) |  |
|  | SPO-FJ |  |  |  |  |  |  |  | 1 |  |  |  | -0.06  (0.77) |

**FIGURE S2** Spearman’s Correlation of MCS, MU, GPR and Delivery time of the four plans. The correlations was described in correlation coefficients (corresponding *p*)

**TABLE 1** The objectives and constraints of the plans in uRT-TPOIS TPS.

| The ROIs | ROIs Property | Constraints |  | Priority |
| --- | --- | --- | --- | --- |
| PGTV/PCTV | PTV | The Minimum Dose | ≥ Prescription dose +80 cGy | 45 |
|  |  | The Minimum DVH | V_Prescription dose +100 cGy_ ≥98% | 50 |
|  |  | The Maximum Dose | ≤ 110% *Prescription dose | 30 |
|  |  | Efficient Ring | The PD fall from 102% to 50% in 5%/mm | 10 |
| SpinalCord | Organ | The Maximum Dose | ≤ 30 Gy | 10 |
| Rectum |  | The Maximum DVH | V45Gy ≤ 55 % | 10 |
|  |  | The Maximum DVH | V40 Gy ≤ 65% | 10 |
| Bladder | Organ | The Maximum DVH | V45Gy ≤ 55 % | 10 |
| Kidney L/R | Organ | The Maximum DVH | V45Gy ≤ 18 % | 10 |
| SmallBowel | Organ | The Maximum Dose | ≤ 50 Gy | 10 |
|  |  | The Maximum EUD | ≤ 23 Gy (α=1) | 5 |
| Colon | Organ | The Maximum Dose | ≤ 60 Gy | 5 |
|  |  | The Maximum EUD | ≤ 25 Gy (α=1) | 5 |
| FemoralHead L/R |  | The Maximum DVH | V30Gy ≤ 15% | 5 |
| PelvicBone | Organ | The Maximum DVH | V40Gy ≤ 30 % | 5 |
|  |  | The Maximum DVH | V30Gy ≤ 60 % | 5 |
|  |  | The Maximum DVH | V20Gy ≤70 % | 5 |
|  |  | The Maximum DVH | V10Gy ≤80 % | 5 |
| Body | External | Single Value | ≤ 63 Gy | 30 |

Note: 1) ROI: Region of Interest; 2) PD: Prescription dose; 3) DVH: Dose-Volume Histogram. The exact values varied slightly depending on the patient.

**TABLE 2**  OARs difference between fixed jaw plans and no fixed jaw plans with different PCTV width (15.5cm as the cutoff)

| PCTV | ≤15.5cm | | ＞15.5cm | | ≤15.5cm | | ＞15.5cm | |
| --- | --- | --- | --- | --- | --- | --- | --- | --- |
| Width | FMO-FJ | FMO-NFJ | FMO-FJ | FMO-NFJ | SPO-FJ | SPO-NFJ | SPO-FJ | SPO-NFJ |
| Bladder |  |  |  |  |  |  |  |  |
| D_mean_ (Gy) | 39.95±1.86 | 39.93±1.93 | 40.66±2.77 | 40.48±2.96 | 38.97±2.5^*^ | 40.41±2.19 | 39.14±3.68^*^ | 41.83±3.67 |
| V_30Gy_(%) | 0.86±0.07 | 0.86±0.08 | 0.88±0.11 | 0.88±0.12 | 0.8±0.10^*^ | 0.88±0.10 | 0.8±0.15^*^ | 0.89±0.12 |
| V_40Gy_(%) | 0.56±0.10 | 0.56±0.10 | 0.59±0.13 | 0.58±0.14 | 0.53±0.10^*^ | 0.58±0.11 | 0.55±0.13^*^ | 0.64±0.16 |
| Rectum |  |  |  |  |  |  |  |  |
| D_mean_(Gy) | 43.19±1.83 | 43.09±1.82 | 43.62±1.32 | 43.57±1.38 | 42.87±1.93^*^ | 43.45±2.35 | 43.39±1.48^*^ | 44±1.57 |
| V_40Gy_(%) | 0.78±0.09 | 0.78±0.09 | 0.81±0.08 | 0.81±0.09 | 0.76±0.09^*^ | 0.78±0.09 | 0.79±0.10^*^ | 0.83±0.09 |
| V_45Gy_(%) | 0.56±0.12 | 0.54±0.13 | 0.55±0.08 | 0.54±0.09 | 0.58±0.10 | 0.58±0.13 | 0.56±0.08 | 0.56±0.09 |
| Kidney |  |  |  |  |  |  |  |  |
| L-D_mean_(Gy) | 8.13±5.86^#^ | 8.37±5.87 | 10.99±5.3^#^ | 11.29±5.47 | 8.04±5.76^*^ | 8.3±5.75 | 10.94±5.28^*^ | 11.12±5.31 |
| L-V_18Gy_(%) | 0.11±0.09 | 0.12±0.09 | 0.16±0.10 | 0.16±0.10 | 0.11±0.09^*^ | 0.12±0.09 | 0.16±0.09^*^ | 0.17±0.09 |
| R-D_mean_(Gy) | 9.1±5.77 | 9.07±5.68 | 11.07±5.34^#^ | 11.32±5.39 | 9.16±5.8 | 9.28±5.86 | 11.11±5.34 | 11.25±5.38 |
| R-V_18Gy_(%) | 0.12±0.09 | 0.12±0.09 | 0.14±0.08 | 0.14±0.08 | 0.13±0.09 | 0.14±0.09 | 0.14±0.08^*^ | 0.16±0.08 |
| Spinal cord |  |  |  |  |  |  |  |  |
| D_max_(Gy) | 33.59±2.05 | 33.54±1.86 | 34.64±2.66 | 34.82±2.63 | 32.25±2.99^*^ | 34.56±1.98 | 32.86±3.66^*^ | 35.37±4.45 |
| Femoral Head |  |  |  |  |  |  |  |  |
| L-V_30Gy_(%) | 0.07±0.05 | 0.08±0.06 | 0.15±0.08 | 0.14±0.07 | 0.05±0.04^*^ | 0.1±0.05 | 0.09±0.05^*^ | 0.15±0.07 |
| R-V_30Gy_(%) | 0.04±0.04 | 0.07±0.05 | 0.15±0.08^*^ | 0.15±0.08 | 0.05±0.04 | 0.09±0.06 | 0.1±0.05^*^ | 0.14±0.07 |
| Small intestine |  |  |  |  |  |  |  |  |
| V_40Gy_(cc) | 70.05±45.73 | 70.32±46.29 | 84.14±54 | 85.5±56 | 69.82±45.62 | 71.77±47.93 | 79.23±50.88^*^ | 85.1±54.29 |
| V_30Gy_(cc) | 170.53±82.44 | 168.84±85.22 | 230.68±125.2^#^ | 240.26±127.5 | 162.75±81.8^*^ | 172.9±87.24 | 209.4±115.23^*^ | 243.16±131.33 |
| Colon |  |  |  |  |  |  |  |  |
| V_40Gy_(cc) | 43.61±37.41 | 43.41±37.83 | 69.21±34.72 | 70.35±36.36 | 43.02±37.56 | 44.54±39.58 | 66.26±32.84^*^ | 73.96±38.16 |
| V_30Gy_(cc) | 61.55±46.06 | 64.09±46.44 | 111.13±52.46 | 112.55±55.97 | 62.32±45.56 | 64.76±49.15 | 104.02±49.15^*^ | 110.7±51.41 |
| Pelvic bone |  |  |  |  |  |  |  |  |
| D_mean_(Gy) | 29.24±1.21 | 29.33±1.24 | 30.04±1.82 | 30.12±1.85 | 28.71±1.24^*^ | 29.26±1.25 | 29.25±1.82^*^ | 30.19±1.79 |
| V_40Gy_(%) | 0.24±0.03 | 0.24±0.03 | 0.27±0.04 | 0.27±0.04 | 0.23±0.02^*^ | 0.25±0.03 | 0.25±0.03^*^ | 0.28±0.04 |
| V_30Gy_(%) | 0.54±0.04 | 0.54±0.04 | 0.56±0.05 | 0.56±0.05 | 0.51±0.04^*^ | 0.53±0.03 | 0.52±0.04^*^ | 0.55±0.04 |
| V_20Gy_(%) | 0.72±0.03 | 0.72±0.04 | 0.73±0.05 | 0.73±0.05 | 0.71±0.03^*^ | 0.72±0.03 | 0.72±0.06^*^ | 0.73±0.05 |
| V_10Gy_(%) | 0.87±0.08 | 0.87±0.08 | 0.87±0.08 | 0.88±0.08 | 0.85±0.09^*^ | 0.86±0.08 | 0.86±0.09^*^ | 0.88±0.08 |

Note: * represents significant difference with SPO-FJ plans (*P*<0.05) ; ^#^ represents significant difference with FMO-FJ plans (*P*<0.05)
